# Assessing cognitive impairment in HIV-infected: a comparative study of international HIV Dementia Scale, HIV Dementia Scale Italian version and Montreal cognitive assessment in clinical practice

**DOI:** 10.1007/s13365-025-01248-9

**Published:** 2025-02-28

**Authors:** Maristella Belfiori, Francesco Salis, Camilla Podda, Lorenzo Stanisci, Benedetta Puxeddu, Francesco Ortu, Paola Piano, Stefano Del Giacco, Antonella Mandas

**Affiliations:** 1 Department of Medical Sciences and Public Health, University of Cagliari, Cagliari, 09042 Italy; 2 Department of Biomedical Sciences, University of Cagliari, Cagliari, 09042 Italy; 3 University Hospital “Azienda Ospedaliero-Universitaria” of Cagliari, Cagliari, 09042 Italy

**Keywords:** HIV, Neurocognitive disorders, Cognitive dysfunction, Outpatients

## Abstract

The combination of antiretroviral therapy (cART) and preventive measures has significantly enhanced the management of Human Immunodeficiency Virus (HIV) infection. However, HIV-associated neurocognitive disorders (HAND) remain a challenge. This study aims to compare cognitive impairment (CI) assessments in people living with HIV/AIDS (PLWHA) using the International HIV Dementia Scale (IHDS), HIV Dementia Scale-Italian Version (HDS-IT) and MoCA (Montreal Cognitive Assessment), while also identifying significant associations. The cross-sectional study encompassed 294 outpatient PLWHA (median age: 57) on cART. Participants underwent cognitive, functional, and depression assessments, laboratory testing and CNS Penetration-Effectiveness (CPE) index assessment. IHDS, HDS-IT and MoCA identified CI in different proportions of PLWHA. Factors such as age, education level, infection duration, and substance use were associated with CI. The IHDS score (OR 0.79) and Level CD4 + T-lymphocytes nadir (OR 0.99) demonstrated independent and negative associations with the CPE-index. IHDS and MoCA tests appear to be useful for detecting CI in outpatient settings, enabling healthcare providers to conduct initial evaluations of PLWHA. IHDS assessment may be used for detecting CI related to high CPE regimens, while the MoCA provides a comprehensive assessment, also in domains not studied by IHDS. However, further research is needed to confirm these findings and refine their clinical applicability.

## Introduction

Over the past decade, significant progress has been made in controlling Human Immunodeficiency Virus (HIV) infection through an improved combination of antiretroviral therapy (cART) and preventive measures like pre-exposure prophylaxis (de la Mora et al. 2024; Keng et al. 2023). In 2021, Botswana, a country with one of the highest global HIV prevalences (where one in five adults is living with HIV), successfully met the 95-95-95 targets. These targets, introduced in 2014 and updated in 2020 by the Joint United Nations Programme on HIV/AIDS (acquired immunodeficiency syndrome), aim to diagnose HIV in 90% of people living with HIV, ensure that 90% of those diagnosed are on treatment, and achieve viral suppression in 90% of those on treatment (Mine et al. 2024; Bai et al. 2020).

Despite these advances, HIV-associated neurocognitive disorders (HAND) remain a persistent public health challenge among people living with HIV/AIDS (PLWHA) (Saylor et al. 2016; McArthur et al. 2010). HAND encompasses a spectrum of neurocognitive impairments, including asymptomatic neurocognitive impairment (ANI), HIV-associated mild neurocognitive disorder (MND) and HIV-associated dementia (HAD) (Moschopoulos et al. 2024; Elbirt et al. 2015). Since the introduction of cART, the prevalence of HAD has decreased from approximately 20% among PLWHA to less than 5% (Hirsch et al. 2022). However, nearly half of PLWHA, particularly those aged over 50 years, present ANI or MND (Zamudio-Rodríguez et al. 2018).

Clinically, HAND predominantly exhibits a subcortical cognitive profile (Antinori et al. 2007). The diagnosis of HAND requires an assessment of at least five domains of neurocognitive functioning, known to be affected by HIV infection, including deficits in the speed of information processing, attention/working memory, episodic memory, motor skills, sensory perception, language and impaired executive functioning (Antinori et al. 2007). Among these, slowed information processing is one of the most prevalent cognitive abnormalities observed in HAND (Cysique et al. 2004; Schouten et al. 2011). Attention and working memory functions are closely related: the ability to create a memory for temporary processing and to retain the information depends on attention functions, which leads to their simultaneous presentation (Schouten et al. 2011). Therefore, impaired fluency is the most frequent language deficit, although it may be associated with mental slowness or executive dysfunction, the latter of which consists of deficits in reasoning, planning, problem-solving and task-switching (Schouten et al. 2011; Dawes et al. 2008).

Older age, advanced World Health Organization clinical staging, and low nadir CD4 count represent significant risk factors for HAND (Heaton et al. 2010; Mohamed et al. 2020). Indeed, HAND remains prevalent in older PLWHA, where age could be related to neurodegenerative disorders like Alzheimer’s disease, leading to an overlap with HAND (Flatt et al. 2023; Nightingale et al. 2023).

Several cognitive screening tools have been developed to facilitate early detection and management of HAND. Among these, the International HIV Dementia Scale (IHDS) and the HIV Dementia Scale (HDS) are specifically designed to assess cognitive impairment (CI) in PLWHA, due to their focus on the subcortical cognitive deficits (Sacktor et al. 2005). Moreover, other screening tools originally developed for broader neurological conditions, such as the Montreal Cognitive Assessment (MoCA), have been adapted and increasingly utilized in the cognitive assessment of PLWHA (Fazeli et al. 2017).

While cART has been instrumental in suppressing HIV replication, the persistence of HAND in cART era suggests the potential neurotoxicity of antiretroviral drugs (Akay et al. 2014; Underwood et al. 2015). According to literature, antiretrovirals have been shown to be toxic to the peripheral neurons in vitro and in vivo, due to alterations in lipid and protein metabolism, mitochondrial damage, and oxidative stress (Power et al. 2009). Recent evidence suggests that the iatrogenic damage might be extended to the Central Nervous System (CNS): oxidative stress, mitochondrial dysfunction, and neuronal damage, induced by cART, may potentially contribute to the pathogenesis of HAND (Akay et al. 2014).

The CNS Penetration-Effectiveness (CPE) index, developed to evaluate antiretroviral drug penetration into the CNS (Avedissian et al. 2020), has shown conflicting results in its association with neurocognitive outcomes. In some studies, it was initially found that higher CPE scores correlated with better neurocognitive functioning, especially in regimens with more than three antiretroviral drugs (Smurzynski et al. 2011). However, recent studies have paradoxically linked higher CPE scores to poorer cognitive performance, despite generally being associated with better control of cerebrospinal fluid viral replication (Marra et al. 2009; Caniglia et al. 2014).

Given the ongoing prevalence of HAND and the potential role of cART in its persistence, this study aims to evaluate the comparative analysis of IHDS, HIV Dementia Scale-Italian Version (HDS-IT) and MoCA assessments. Additionally, the study aims to identify and analyze significant correlations between these screening methods and demographic, pharmacological, and laboratory variables, thereby contributing to the optimization of diagnostic strategies for HAND in the cART era.

## Materials and methods

The cross-sectional study involved participants who were assessed at the Immunology Service of the University Hospital of Monserrato in Cagliari. Italy, from November 2022 to July 2023. The study aims to examine potential differences in assessing cognitive impairment between IHDS, HDS-IT and MoCA in PLWHA and to identify significant associations. The inclusion criteria encompassed individuals aged 18 years or older with confirmed HIV infection, who underwent assessment through the IHDS, HDS-IT and MoCA. Subjects who did not furnish informed consent were excluded from the study.

### Clinical and laboratory assessment

Upon initial assessment, we obtained demographic information, the infection’s transmission mode and ongoing cART details. We divided the current therapy into six distinct pharmacological categories, namely nucleoside reverse transcriptase inhibitors (NRTIs), nucleotide analogues (NtRTIs), integrase inhibitors (INIs), non-nucleoside reverse transcriptase inhibitors (NNRTIs), protease inhibitors (PIs) and CCR5 inhibitors. Furthermore, we defined the penetrative ability of antiretroviral drugs into the CNS through the CPE-index. Laboratory analyses included the assessment of blood CD4 + T-lymphocyte counts (both current levels and nadir, indicating the lowest recorded value), the ratio of CD4 + to CD8 + lymphocytes, and HIV RNA load (both current levels and zenith. indicating the highest recorded value), hemoglobin (Hb), white blood cell count (WBC), platelet count (PLT), creatinine, aspartate aminotransferase (AST), alanine aminotransferase (ALT), alkaline phosphatase (ALP), gamma-glutamyl transferase (GGT), total bilirubin, total protein, low-density lipoprotein (LDL), ferritin, vitamin B12, folate and C-reactive protein (CRP).

### Cognitive. Affective and functional assessment

During the enrollment process, participants underwent a comprehensive clinical evaluation, which included cognitive, functional, and depression assessments.

Cognitive impairment was assessed using the following instruments:

- IHDS (Sacktor et al. 2005; Rosca et al. 2021), a rapid and accessible cognitive screening tool, particularly advantageous for non-neurologists due to its simplicity and reduced training requirements. Developed to minimize cultural biases, the IHDS replaces tasks that could disadvantage individuals from non-Western backgrounds, such as alphabet writing and cube copying, with motor speed and sequencing tasks. The IHDS includes memory registration of four words for later recall, timed finger tapping, an adapted Luria’s fist-edge-palm test for hand sequence and recall of the four words after two minutes. The test is scored with a maximum total of 12 points, with a score of ≤ 10 suggesting the need for further cognitive evaluation.
- HDS-IT (Power et al. 1995; Montanucci et al. 2021), rapid cognitive screening for HAND, particularly for HAD, which takes around 10 min to administer. It comprises four subtests that evaluate different cognitive domains: attention (anti-saccadic eye movements), psychomotor speed (timed alphabet writing), memory/recall (following memory registration), and construction (cube drawing). The original HDS validation demonstrated 80% sensitivity and 91% specificity for detecting HAD using a cut-off score of ≤ 10. The Italian version of the HDS has adapted the scale for linguistic and cultural differences, with a maximum score of 16 and a recommended cut-off of ≤ 11, which correlates with good sensitivity (0.70) and specificity (0.82).
- MoCA (Salis et al. 2024; Rosca et al. 2019), another cognitive screening tool, which takes approximately 10–15 min to complete. It can be administered by lay personnel, is easy to score, and requires a simple correction for years of education. Commonly used to measure global cognitive function in both clinical and research contexts, the test consists of 30 items, that encompass eight cognitive domains, including short-term memory, attention, working memory, and frontal-executive functions, commonly affected in HIV patients. MoCA is scored out of 30 points and is adjusted for education level: a score of 25 or below indicates a potential cognitive impairment.

Depressive symptoms were assessed by the Patient Health Questionnaire-two items (PHQ-2) (Villarreal-Zegarra et al. 2023), a brief screening tool designed to assess depressive symptoms and anhedonia experienced over the past two weeks, through the first two items of PHQ-9. The PHQ-2 score ranges from 0, indicating no depressive symptoms, to 6, suggesting daily depressive symptoms. A score of 3 or higher is indicative of a depressive mood.

The functional status was assessed using the Activities of Daily Living (ADL) and the Instrumental Activities of Daily Living (IADL) scales. The ADL scale (Hyejin et al. 2021) evaluates a person’s independence in common daily activities such as personal hygiene, dressing, using the toilet, walking, sitting, standing, lying down, climbing stairs, continence, and eating. It ranges from 0 (complete dependence) to 6 (complete autonomy). The IADL scale (Goodkin et al. 2017) assesses a person’s ability to perform instrumental activities necessary for life at home and in the community, such as using the telephone, managing finances, shopping, food preparation, housekeeping, laundry, transportation, and medication. The score ranges from 0 (complete dependence) to 8 (complete autonomy).

### Statistical analysis

Because the variables examined were not normally distributed, quantitative variables were presented as median and interquartile ranges (IQR) or percentages (%), as appropriate. The Mann-Whitney U test was used for continuous variables, and Pearson’s chi-squared test was used for categorical variables. The correlation between cognitive screening tools was examined using Spearman’s rank correlation coefficient (ρ). A p-value of less than 0.05 was considered statistically significant. The multivariate analysis was conducted using a logistic regression-stepwise, and variables with p greater than 0.1 were excluded from the model: its results, along with their standard errors and odds ratios (ORs), were assessed using the area under the ROC curve (AUC). The Venn diagram was used to show the overlap of cognitive impairment detected by screening tools. The results are presented with corresponding p-values and 95% confidence intervals (95%CI). The statistical analysis was conducted using MedCalc software Ltd. (Version 22.030. Ostend. Belgium).

## Results

The study recruited 294 PLWHA, aged 30 years or older (median: 57; IQR 51–60). As shown in Table 1, more than half (65.65%) were men and the median age at infection was 25 years.

**Table 1:** Demographic and clinical characteristics of the sample

Variable	
*Age (years)*	57 (51–60)
*Male*,* n (%)*	193 (65.65)
*Female*,* n (%)*	101 (34.35)
*Education (years)*	11 (8–13)
*Primary or below*	15 (5.10)
*Lower secondary school*	112 (38.10)
*Upper secondary school or above*	167 (56.80)
*Years of infection*	25 (13–31)
*Routes of trasmission*,* n (%)*	
*Homosexual contacts*	70 (23.80)
*MSM*	68 (97.14)
*IVDA*	105 (35.71)
*Heterosexual transmission*	106 (36.05)
*Transfusion transmission*	4 (1.36)
*Not specified/Other*	31 (10.54)
*Antiretroviral therapy*,* n (%)*	
*NRTI*	225 (76.53)
*NNRTI*	102 (34.69)
*NtRTI*	196 (66.67)
*PI*	46 (15.65)
*INI*	189 (64.29)
*CCR5i*	10 (3.40)
*CPE Index*	3 (3–4)
*CD4 + T-lymphocytes (cells/µL)*	742 (534–985)
*CD4+/CD8 + T-lymphocytes ratio*	1.04 (0.65–1.48)
*CD4 + T-lymphocytes nadir (cells/µL)*	250.50 (118.00-389.00)
*HIV-RNA (copies/µL)*	0 (0–0)
*HIV-RNA Zenith (copies/µL)*	67,357 (16616–166400)
*Hb (g/dL)*	14.50 (13.30–15.60)
*WBCs (×10* ^*3*^ */µL)*	6.29 (5.26–7.48)
*PLTs (×10* ^*3*^ */µL)*	224 (178–226)
*Creatinine (mg/dL)*	1.03 (0.91–1.16)
*AST (U/L)*	22 (18–28)
*ALT (U/L)*	22 (15–29)
*GGT (U/L)*	24 (17–36)
*Total bilirubin (mg/dL)*	0.61 (0.46–0.83)
*Total protein (g/dL)*	7.20 (6.90–7.50)
*LDL (mg/dL)*	108.50 (85.00-128.00)
*Ferritin (µg/L)*	101.60 (56.30-181.30)
*Vitamin B*_*12*_ *(ng/mL)*	459 (357–552)
*Folate (ng/mL)*	8.65 (6.18–12.80)
*IHDS*	9.50 (8.00–11.00)
*HDS-IT*	13.50 (11.00–15.00)
*MoCA (adjusted for ages of school)*	25 (23–27)
*PHQ-2*	0 (0–2)
*ADL*	6 (6–6)
*IADL*	8 (8–8)

Data are presented as n (%) or median (IQR)

Abbreviations: **ADL**, Activities of Daily Living; **ALT**, Alanine aminotransferase; **AST**, Aspartate aminotransferase; **CCR5i**, CCR5 inhibitor; **CPE index**, Central nervous system penetration-effectiveness index; **GGT**, Gamma-Glutamyl Transferase; **Hb**, Hemoglobin; **HDS-IT**, Hiv Dementia Scale- Italian version; **HIV**, Human Immunodeficiency Virus; **IADL**, Instrumental Activities of Daily Living; **IHDS**, International HIV Dementia Scale; **INI**, Integrase inhibitor **IQR**, Interquartile range; **IVDA**, Intravenous Drug Users; **LDL**, Low-Density Lipoproteins; **MoCA**, Montreal Cognitive Assessment; **MSM**, Men who have Sex with Men; **NNRTI**, Non-Nucleoside Reverse Transcriptase Inhibitor; **NRTI**, Nucleoside Reverse Transcriptase Inhibitor; **NtRTI**, Nucleotide Reverse Transcriptase Inhibitor; **PHQ-2**, Patient Health Questionnaire– two items; **PI**, Protease inhibitor; **PLT**, Platelets; **WBC**, White Blood Cell

The majority of the sample (56.80%) presented an education level of 9 years or more, with a global median of 11 (8–13).

In HIV acquisition, heterosexual contact (36.05%) was the primary route of transmission among the sample, especially among females (66.33% vs. 20.2%, χ^2^ = 60.99, *p* < 0.0001); among males, intravenous drug users (IVDA) (40.93% vs. 25.7, χ^2^ = 6.64, *p* = 0.0100), followed by men who have sex with men (MSM) (35.23% vs. 2.0%, χ^2^ *=* 40.28, *p* < 0.0001), prevailed.

Regarding cART, the predominant drug classes utilized included NRTIs, at a rate of 76.53%, and INIs, at a rate of 64.29%. Furthermore, 203 participants (69.05%) administered a regimen of three or more drug classes. A total of 21 distinct cART combinations were identified within the cohort, highlighting the diverse treatment regimens used (Table 2). According to the CPE Index, the median score was 3 (3–4). Based on the obtained scores, a high CNS penetrating regime was defined as CPE ≥ 6. The median CD4 + T-lymphocytes nadir was 250.5 (118–389) and the HIV- RNA Zenith was 67,357 copies/µL (16616–166400). The sample demonstrated preserved independence in ADLs and IADLs, as indicated by median scores (6/6 for ADLs and 8/8 for IADLs) and interquartile ranges (6–6 for ADLs and 8–8 for IADLs).

**Table 2:** cART combinations

Number of patients	cART combinations
*2*	NtRTI + *PI + INI*
*2*	*CCR5i + INI*
*20*	INI
*4*	PI + *CCR5i*
*10*	PI + INI
*2*	NNRTI + CCR5i + INI
*22*	NNRTI + INI
*3*	NNRTI + PI
*4*	NNRTI + PI + INI
*1*	NRTI + NtRTI
*3*	NRTI + NtRTI + CCR5i
*98*	NRTI + NtRTI + INI
*19*	NRTI + NtRTI + PI
*1*	NRTI + NtRTI + PI + CCR5i
*1*	NRTI + NtRTI + PI + INI
*28*	NRTI + INI
*1*	NRTI + PI
*1*	NRTI + PI + INI
*70*	NRTI + NNRTI + NtRTI
*1*	NRTI + NNRTI + NtRTI + INI
*1*	NRTI + NNRTI + NtRTI + INI + PI

Abbreviations: **CCR5i**, CCR5 inhibitor; **INI**, Integrase inhibitor; **NNRTI**, Non-Nucleoside Reverse Transcriptase Inhibitor; **NRTI**, Nucleoside Reverse Transcriptase Inhibitor; **NtRTI**, Nucleotide Reverse Transcriptase Inhibitor; **PI**, Protease inhibitor

The majority of PLWHA, specifically 189 (64.28%) demonstrated cognitive impairment as per the IHDS. Additionally, 76 (25.85%) and 171 (58.16%) individuals were classified as cognitively impaired according to the HDS-IT and MoCA (adjusted for ages of school), respectively.

**Fig. 1:**
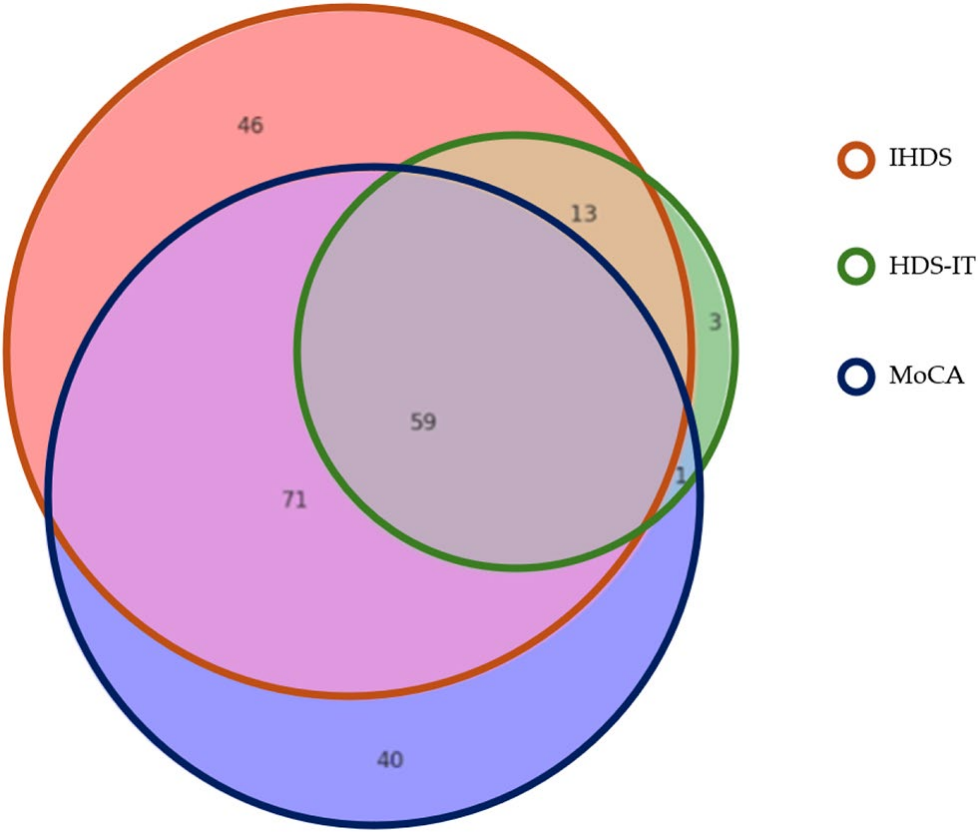
Overlap of cognitive impairment detected by IHDS, HDS-IT and MoCA- Venn diagram. Figure Note: Subjects with cognitive impairment intercepted by the IHDS are indicated in area outlined in orange, those intercepted by HDS-IT are in area outlined in green, and those intercepted by MoCA are in area outlined in blue. Abbreviations: **HDS-IT**, HIV Dementia Scale Italian version; **IHDS**, International HIV Dementia Scale; **MoCA**, Montreal Cognitive Assessment

As illustrated in Fig. 1, the Venn diagram shows that IHDS or MoCA also identified most of the cognitive impairment determined by the HDS-IT among PLWHA.

**Fig. 2:**
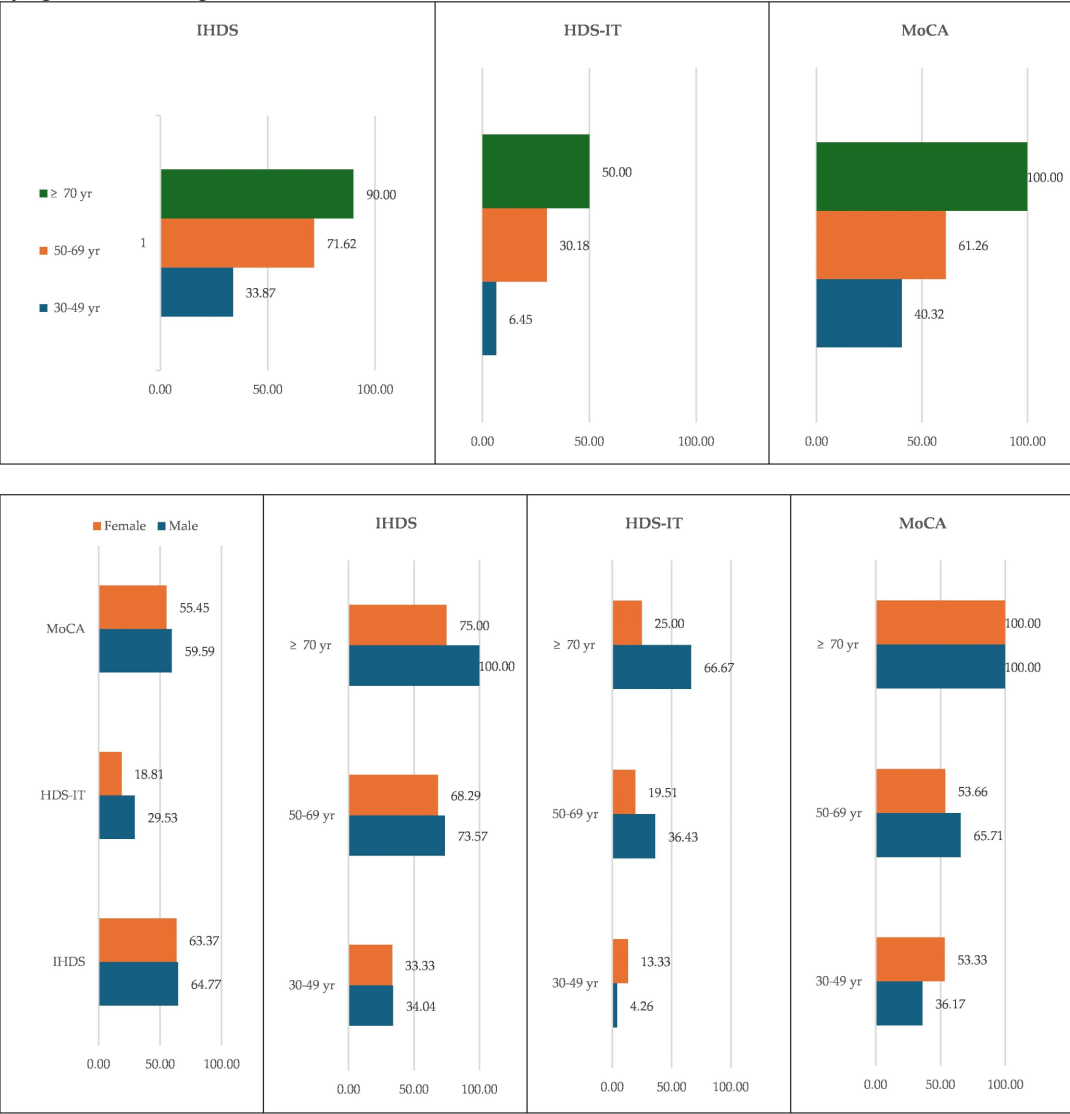
Prevalence of cognitive impairment as detected by IHDS, HDS-IT and MoCA in PLWHA, stratified by age brackets and gender. Abbreviations: **HDS-IT**, HIV Dementia Scale Italian version; **IHDS**, International HIV Dementia Scale; **MoCA**, Montreal Cognitive Assessment

The sample was divided into three age categories (30–49 years, 50–69 years and 70 years or older) and stratified by gender (Fig. 2).

As age advanced, an increasing prevalence of cognitive impairment was detected, with the highest frequency recorded through the MoCA assessment. In males, cognitive impairment was more frequently observed using various cognitive screening tools, across all age brackets, except in the 30–49 age group, where females were more cognitively impaired, using the HDS-IT and MoCA assessments. Additionally, in the age group of 70 years and above, males and females demonstrated cognitive impairment according to the MoCA assessment. But, after conducting the Mann-Whitney test (Table 3), no significant differences between females and males were observed using HIDS (*p* = 0.5520), HDS-IT (*p* = 0.2269) and MoCA assessments (*p* = 0.3141). Females presented a higher median score on PHQ-2 (*p* = 0.0143) and a longer infection duration (*p* = 0.0017). Furthermore, males displayed lower level of CD4 + T-lymphocytes (*p* = 0.0073) and CD4+/CD8 + T-lymphocytes ratio (*p* < 0.0001).

**Table 3:** Characteristics of PLWHA according to gender

	Females (*n* = 101)	Males (*n* = 193)	*p*
*Age (years)*	58 (53–60)	56 (50–60)	0.1265
*Years of infection*	27 (21–31)	20 (12–30)	**0.0017**
*Education (years)*	10 (8–13)	11 (8–13)	0.4004
*CPE Index*	3 (3.00-4.25)	3 (3–3)	0.76
*CD4 + T-lymphocytes (cells/µL)*	826 (581–1091)	716 (494.25-915.25)	**0.0073**
*CD4+/CD8 + T-lymphocytes ratio*	1.31 (0.89–3.52)	0.89 (0.59–1.47)	**< 0.0001**
*CD4 + T-lymphocytes nadir (cells/µL)*	234 (118-350.50)	257 (117.75–398.50)	0.3658
*HIV-RNA (copies/µL)*	0 (0–0)	0 (0–0)	0.5513
*HIV-RNA Zenith (copies/µL)*	46,300 (16504.50-162050)	73,000 (16875–166400)	0.2940
*Hb (g/dL)*	13.80 (12.96–14.60)	15 (13.60–15.90)	**< 0.0001**
*WBCs (×10* ^*3*^ */µL)*	6.09 (4.96–7.42)	6.41 (5.38–7.59)	0.1135
*PLTs (×10* ^*3*^ */µL)*	230 (181-282.75)	219 (177–264)	0.3548
*Creatinine (mg/dL)*	0.93 (0.83–1.02)	1.09 (0.98–1.21)	**< 0.0001**
*AST (U/L)*	21 (17–24)	22 (19–29)	**0.0036**
*ALT (U/L)*	18 (13–26)	23 (16.75–32.25)	**< 0.0001**
*GGT (U/L)*	20 (15–28)	26 (19–40)	**< 0.0001**
*Total bilirubin (mg/dL)*	0.57 (0.44–0.70)	0.64 (0.47–0.88)	**0.0236**
*Total protein (g/dL)*	7.20 (6.90–7.40)	7.20 (6.90–7.50)	0.4887
*Albumin (g/dL)*	4.24 (4.04–4.39)	4.29 (4.09–4.48)	0.0917
*LDL (mg/dl)*	109 (84–128)	108 (88.50-127.25)	0.8352
*Ferritin (µg/l)*	67.40 (42.73-105.15)	128.80 (73.38-205.53)	**< 0.0001**
*Vitamin B12 (ng/ml)*	466 (373.25- 594.25)	451 (352-546.75)	0.1877
*Folate (ng/ml)*	9.64 (6.80-13.25)	8.20 (5.90-12.31)	0.0692
*IHDS*	10 (8–11)	9.50 (8–11)	0.5520
*HDS-IT*	14 (12–15)	13.50 (10.50–15)	0.2269
*MoCA (adjusted for ages of school)*	25 (23–27)	25 (22–27)	0.3141
*PHQ-2*	1 (0–3)	0 (0–2)	**0.0143**

Abbreviations: **ALT**, Alanine aminotransferase; **AST**, Aspartate aminotransferase; **CPE index**, Central nervous system penetration-effectiveness index; **GGT**, Gamma-Glutamyl Transferase; **Hb**, Hemoglobin; **HDS-IT**, Hiv Dementia Scale- Italian version; **HIV**, Human Immunodeficiency Virus; **IHDS**, International HIV Dementia Scale; **LDL**, Low-Density Lipoproteins; **MoCA**, Montreal Cognitive Assessment; **PHQ-2**, Patient Health Questionnaire– two items; **PLT**, Platelets; **WBC**, White Blood Cell

Furthermore, we assessed PLWHA and stratified based on normal and pathological scores using cognitive screening tools (Table 4). Individuals with poor scores on the IHDS (*p* < 0.0001) and HDS-IT (*p* = 0.0003) showed a longer infection duration, as compared to those with normal scores. A lower level of education characterized cognitively impaired patients, detected by IHDS (*p* < 0.0001), HDS-IT (*p* < 0.0001) and MoCA (*p* = 0.0073) assessments. PLWHA with pathologic scores on IHDS (*p =* 0.0001), HDS-IT (*p* < 0.0001) and MoCA (*p* = 0.0005) were older than PLWHA with normal scores. A larger share of patients with pathological scores on IHDS (43.4% vs. 21.9%, χ^2^ = 13.52, *p* = 0.0002) and HDS-IT (59.2% vs. 27.5%, χ^2^ = 24.56, *p* < 0.0001) were IVDA. Poor IHDS performers presented a normal median score on HDS -IT (*p* < 0.0001) and vice versa (good HDS-IT performers presented a pathological score on IHDS, *p* < 0.0001). Indeed, approximately 40% of the 189 patients with pathological IHDS scores exhibited normal scores on the HDS-IT (39.79%), although Spearman’s correlation intercepted a positive and strong correlation between the two screening tools (*r* = 0.633; *p* < 0.0001). Furthermore, good HDS-IT (*p* < 0.0001) performers showed pathologic scores on MoCA and vice versa (poor MoCA performers presented normal median score on HDS, *p* < 0.0001), with a moderate correlation (*r* = 0.54; *p* < 0.0001).

**Table 4:** Characteristics of PLWHA according to IHDS, HDS-IT and MoCA

	IHDS		*p*	HDS-IT		*p*	MoCA		*p*
	Not- impaired	Impaired		Not- impaired	Impaired		Not- impaired	Impaired	
	> 10	≤ 10		> 11	≤ 11		≥ 26	< 26	
	***(*** *n* ** = 105)**	***(*** *n* ** = 189)**		***(*** *n* ** = 218)**	***(*** *n* ** = 76)**		***(*** *n* ** = 123)**	***(*** *n* ** = 171)**	
*Age (years)*	53 (44–59)	58 (54–61)	**0.0001**	56 (48–59)	59 (54–62)	**< 0.0001**	54 (46–60)	58 (53–61)	**0.0005**
*Years of infection*	16 (11–26)	27 (17–32)	**< 0.0001**	21 (12–29)	28 (23–33)	**0.0003**	23 (13–31)	26 (13–31)	0.633
*Education (years)*	13 (8–16)	8 (8–13)	**< 0.0001**	13 (8–14)	8 (8–9)	**< 0.0001**	13 (8–13)	9 (8–13)	**0.0073**
*CPE Index*	3 (3–3)	3 (3–5)	**0.0408**	3 (3–3)	3 (3–5)	0.31	3 (3–3)	3 (3–4)	0.97
*CD4 + T-lymphocytes (cells/µL)*	802 (546.75–1040.00)	717 (502.25–955.00)	0.23	782.50 (540–1014)	687.50 (493–863)	**0.0429**	790 (528.25-1053.50)	722 (539.25–940.00)	0.31
*CD4+/CD8 + T-lymphocytes ratio*	1.04 (0.74 -1087.50)	1.04 (0.63–1.66)	0.73	1.10 (0.70–1.93)	0.91 (0.49–1.52)	**0.0199**	1.07 (0.67–1.93)	1.02 (0.65–1.68)	0.51
*CD4 + T-lymphocytes nadir (cells/µL)*	275 (139.25- 422.25)	238 (113.00- 375.50)	0.06	269.50 (132–398)	191.50 (83–344)	**0.0186**	258 (118.00-400.25)	248 (119.50–374.00)	0.48
*HIV-RNA (copies/µL)*	0 (0–0)	0 (0–0)	0.68	0 (0–0)	0 (0–0)	0.20	0 (0–0)	0 (0–0)	0.14
*HIV-RNA Zenith (copies/µL)*	79,200 (16504.50- 168875)	62,100 (16674–163625)	0.64	67,110 (17600–164000)	67,357 (11562.50- 178500)	0.49	69,300 (16670–163250)	64,920 (16200–174425)	0.68
*Hb (g/dL)*	14.5 (13.08–15.30)	14.5 (13.40-15.63)	0.41	14.5 (13.40–15.60)	14.5 (12.70–15.60)	0.57	14.6 (13.13–15.58)	14.4 (13.30–15.60)	0.78
*WBCs (×10* ^*3*^ */µL)*	6.05 (5.01–7.41)	6.47 (5.38–7.55)	**0.0470**	6.22 (5.18–7.42)	6.84 (5.38–7.64)	0.07	6.22 (5.27–7.58)	6.43 (5.22–7.41)	0.65
*PLTs (×10* ^*3*^ */µL)*	229 (189.00- 278.50)	221 (175.50–262)	0.17	221 (177–266)	230 (183.50–263.00)	0.7641	228 (176.25–278.00)	221 (180.75- 254.75)	0.45
*Creatinine (mg/dL)*	1.04 (0.93–1.16)	1.02 (0.91–1.16)	0.40	1.03 (0.90–1.16)	1.04 (0.93–1.19)	0.60	1.03 (0.92–1.16)	1.03 (0.90–1.18)	0.88
*AST (U/L)*	21 (18.00-26.25)	22 (18.00-28.25)	0.3768	21 (18–26)	23.50 (20.00- 29.50)	**0.0303**	21 (18–26)	22 (18–29)	0.2294
*ALT (U/L)*	20 (15–30)	22 (15–29)	0.7723	22 (15–29)	21.50 (17.00–29.00)	0.2993	21.00 (15.00–29.00)	23.00 (15.25–30.75)	0.3447
*GGT (U/L)*	21 (16.75–32.25)	25 (18–37)	0.1017	23 (16–33)	29.50 (18.50–55.50)	**0.0048**	22 (17–30)	26 (18–39)	0.0688
*Total bilirubin (mg/dL)*	0.59 (0.46–0.82)	0.62 (0.45–0.84)	0.6816	0.62 (0.46–0.85)	0.57 (0.42–0.73)	0.2091	0.63 (0.48–0.87)	0.57 (0.44–0.80)	0.0691
*Total protein (g/dL)*	7.10 (6.70–7.40)	7.20 (7.00-7.53)	**0.0043**	7.10 (6.80–7.40)	7.40 (7.10–7.60)	**0.0004**	7.20 (6.83–7.48)	7.20 (7.00-7.50)	0.1881
*LDL (mg/dL)*	111 (84–128)	107 (85.75-127.25)	0.6769	111 (85–130)	103 (85.00-117.50)	0.1768	111 (90.50–126.00)	107 (83.25–128.00)	0.2385
*Ferritin (µg/L)*	99.70 (56.60–174.73)	106.20 (56.30-184.53)	0.6346	99.70 (53.90- 174.30)	106.65 (62.65–207.50)	0.1279	107.10 (58.10-173.90)	100.80 (55.33- 183.98)	0.9651
*Vitamin B*_*12*_ *(ng/mL)*	464 (374.25- 592.75)	453 (355-540.50)	0.4497	463 (365–557)	436 (355–544)	0.5474	458 (354.75-550.75)	460 (358.25-555.75)	0.9933
*Folate (ng/mL)*	9.10 (6.70-13.33)	8.40 (5.90-12.31)	0.3758	9.10 (6.40–13.40)	8 (5.70-10.23)	**0.0282**	8.90 (6.79–12.38)	8.40 (5.83–12.90)	0.4666
*IHDS*	/	/		10 (9–11)	7 (6.00-8.75)	**< 0.0001**	10.5 (9-11.50)	9 (13-15.50)	**< 0.0001**
*HDS-IT*	15 (14–16)	12 (10–14)	**< 0.0001**	/	/		14.50 (13- 15.50)	12.50 (10.50–14)	**< 0.0001**
*MoCA (adjusted for ages of school)*	26 (24–28)	24 (21–26)	**< 0.0001**	25 (23–27)	23 (20–25)	**< 0.0001**	/	/	
*PHQ-2*	1 (0–2)	0 (0–2)	0.7480	0 (0–2)	1 (0–3)	0.2187	0 (0-1.75)	1 (0–3)	**0.0058**

Abbreviations: **ALT**, Alanine aminotransferase; **AST**, Aspartate aminotransferase; **CPE index**, Central nervous system penetration-effectiveness index; **GGT**, Gamma-Glutamyl Transferase; **Hb**, Hemoglobin; **HDS-IT**, Hiv Dementia Scale- Italian version; **HIV**, Human Immunodeficiency Virus; **IHDS**, International HIV Dementia Scale; **LDL**, Low-Density Lipoproteins; **MoCA**, Montreal Cognitive Assessment; **PHQ-2**, Patient Health Questionnaire– two items; **PLT**, Platelets; **WBC**, White Blood Cell

In our study, a logistic regression model was developed to assess the impact of various factors on the CPE index, serving as the dependent variable. The independent variables encompassed scores from cognitive and affective screening tools, demographic indicators, transmission routes, as well as immunovirological and hematologic examinations. The IHDS score (OR 0.79, 95% CI 0.68–0.92, *p* = 0.0023) and Level CD4 + T-lymphocytes nadir (OR 0.99, 95% CI 0.99–0.99, *p* = 0.0004) demonstrated independent and negative associations with the CPE-index (AUC: 0.73, standard error: 0.04, 95%CI: 0.68–0.78); other variables were excluded from the model. After conducting the χ^2^ test, it was determined that 87.0% of PLWHA with a high CPE-Index and 62.4% of PLWHA with a non-high CPE-Index exhibited cognitive impairment on IHDS (χ^2^: 5.57, *p* = 0.0183). Another logistic regression analysis was conducted to examine the association between transmission routes and cognitive performance as assessed by the HDS-IT, IHDS and MoCA. In the respective logistic regressions with HDS-IT, IHDS, and MoCA as dependent variables, no significant association was found with homosexual transmission (*p* = 0.3780; *p* = 0.1659; *p* = 0.0840), heterosexual transmission (*p* = 0.6435; *p* = 0.4910; *p* = 0.5262), transfusion transmission (*p* = 0.9981; *p* = 0.9981; *p* = 0.9698), and unspecified/other transmission (*p* = 0.2898; *p* = 0.9068; *p* = 0.3448).

## Discussion

This study provides a comprehensive overview of the cognitive profiles of a representative sample of PLWHA in the cART era. Our findings reveal several insights into the prevalence of CI among this population, sex-specific differences in HIV progression, the role of various biomarkers in understanding the impact of the disease and ART-related neurotoxicity.

The cohort analyzed in this study comprised predominantly male PLWHA, with a median age of 57 years and a prolonged duration of HIV infection. This demographic profile aligns with trends observed over recent decades, where HIV-infected men prevail (Chan et al. 2019), and the median age of PLWHA has notably increased, with a substantial proportion now aged 50 years or older (Gebo and Moore 2004; Patterson et al. 2015). The aging of the HIV-positive population is largely attributable to enhanced life expectancy due to antiretroviral therapy (Hasse et al. 2011). However, this demographic shift introduces a set of distinctive challenges, including a higher incidence of comorbidities such as hypertension, hypertriglyceridemia, and reduced bone mineral density (Onen et al. 2010; Deeks 2011). Additionally, older HIV patients are at an elevated risk of polypharmacy and experience greater susceptibility to toxic effects associated with antiretroviral therapies (Gebo and Moore 2004). The study found a notable prevalence of CI within our cohort, using the IHDS and MoCA screening tools. The lower prevalence detected by HDS, following previous studies, might be attributed to the greater sensitivity of HDS in detecting dementia rather than mild cognitive impairment (Ganasen et al. 2007; Lu et al. 2014), although the use of a higher cut-off (11) (Montanucci et al. 2021). Furthermore, IHDS may be more sensitive in identifying mental slowness, attention/memory deficits and impaired executive functioning (Chan et al. 2019), all associated with HAND. Additionally, while MoCA is a general cognitive screening tool that has been adapted for use in HIV, it has demonstrated good sensitivity (84.2%) in detecting CI in HIV-positive individuals (Fazeli et al. 2017).

Gender differences were also prominent in our study. According to the literature, 66.33% of women contracted HIV infection through heterosexual transmission, while a substantial proportion of men reported homosexual contacts or drug use (Nunes et al. 2010). Women presented a longer duration of infection and higher CD4 counts, aligning with previous research, indicating that women often have better immunological control of the infection, likely due to earlier care entry and the higher CD4 counts at the enter care (Biber et al. 1999), and biological factors such as oestrogen receptor signaling (Griesbeck et al. 2016) and higher circulating interleukin-7 levels, which play a crucial role in T-cell production and homeostasis (Napolitano et al. 2005). However, extended infection in women is also associated with elevated incidences of psychosocial illnesses (Mello et al. 2006).

Our study highlighted the associations between a longer duration of infection with poorer cognitive performance on IHDS and HDS-IT. The data aligns with existing literature: prolonged exposure to HIV, even in the context of cART, can contribute to neurocognitive decline over time (Heaton et al. 2010). Age also emerged as a significant factor, with older patients showing worse cognitive performance across all cognitive assessments. This is expected, as ageing is a well-established risk factor for cognitive decline in both the general population and individuals with HIV (Nightingale et al. 2023; Lam et al. 2021). The compounding effects of ageing and long-term HIV infection may exacerbate cognitive deficits, making it a critical area of concern as the HIV-positive population continues to age. Patients with lower educational attainment were likelier to score poorly across all three cognitive screening tools. This is consistent with the literature, showing a protective effect of higher education against cognitive decline, potentially due to greater cognitive reserve (Alvarez-Tostado et al. 2016). Patients with a history of IVDA were likelier to exhibit pathological cognitive scores on IHDS and HDS, which may be due to the combined neurotoxic effects of both HIV and substance abuse on the brain (Nightingale et al. 2023). Interestingly, the results showed that most patients with pathological IHDS scores had normal HDS-IT scores, and vice versa. The IHDS, for example, includes a motor component that might capture motor-related cognitive deficits that the HDS-IT does not. Conversely, the HDS-IT might focus more on attention functions and easier psychomotor speed tests, which could explain why some patients perform differently across the two tests. Despite these differences, the significant correlation between IHDS and HDS-IT (*r* = 0.633; *p* < 0.0001) indicates that both tools may be valuable in detecting CI, although they could be assessing distinct cognitive domains. Furthermore, the moderate correlation between HDS-IT and MoCA and the observation that patients with good HDS-IT scores often performed poorly on the MoCA suggest that MoCA may capture broader cognitive domains not assessed by HDS-IT or be more sensitive to milder cognitive impairment.

Finally, the analysis of the CPE index revealed a complex relationship between high CPE scores, neurocognitive performance, and CD4 nadir levels. While a higher CPE index is designed to improve HIV suppression in the CNS, it is paradoxically associated with poorer cognitive outcomes, as also reported in other studies (Marra et al. 2009). This may be due to the neurotoxic effects of antiretroviral drugs with high CNS penetration, which could contribute to cognitive decline despite viral suppression (Akay et al. 2014). The IHDS identified a higher proportion of cognitive deficits in individuals receiving high CPE regimens compared to the HDS and MoCA, which may suggest its potential utility in detecting ART-related neurotoxicity. Nevertheless, additional research is necessary to validate this association. In our analysis, logistic regression models did not reveal significant associations between HIV transmission routes and cognitive performance, as evaluated by the IHDS, HDS-IT, and MoCA assessments. These findings are consistent with previous studies (De Francesco et al., 2020), although further research is warranted to explore potential confounding factors that may influence this association.

Several limitations of this study must be acknowledged. Firstly, the cross-sectional nature of this study limits our ability to determine temporal or causal links between the factors examined and the onset of CI. Furthermore, the absence of comprehensive neuropsychological testing represents a limitation and should be integrated into future studies. Also, the sample representative of the CPE index *≥* 6 was smaller than the PLWHA with not-high CPE index.

Moreover, the substantial variability observed in cART regimens within our cohort has resulted in inadequate sample sizes for each specific combination. Consequently, meaningful statistical evaluations of their potential association with CI could not be conducted. Future studies with larger and more homogeneous cohorts may help clarify the impact of specific treatment regimens on cognitive function.

## Conclusions

Our research highlights the link between CI and factors such as age, education level, infection duration and substance use among PLWHA. Due to its ease of administration and time efficiency, the IHDS could be a practical option for initial screening, particularly in cases with high CPE regimens, though further validation is necessary. The MoCA provides a more comprehensive assessment, also in domains not studied by IHDS but relevant for older PLWHA at higher risk for comorbidities like Alzheimer’s disease and cerebrovascular conditions (Fazeli et al. 2017). Therefore, our findings suggest that using IHDS and MoCA may serve as valuable tools for screening CI in outpatient settings.

These assessments enable medical professionals trained in cognitive evaluation to perform initial assessments during routine clinical visits. While these tools facilitate practical routine evaluations, it remains imperative to conduct a comprehensive neuropsychological assessment to confirm cognitive impairment in patients who exhibit poor performance on the IHDS and/or MoCA. Further research is required to validate the efficacy of this approach and to enhance screening protocols for CI in clinical practice.

## Data Availability

Data is available upon reasonable request.

## Abbreviations

AIDS: Acquired Immunodeficiency Syndrome
ADL: Activities of Daily Living
ALT: Alanine aminotransferase
ALP: Alkaline phosphatase
ANI: Asymptomatic Neurocognitive Impairment
AUC: Area Under the ROC Curve
CCR5i: CCR5 inhibitor
CI: Cognitive Impairment
MND: Mild Neurocognitive Disorder
cART: Combination Antiretroviral Therapy
CNS: Central Nervous System
CRP: C-Reactive Protein
CPE: CNS Penetration-Effectiveness
GGT: Gamma-Glutamyl Transferase
HAND: HIV-Associated Neurocognitive Disorders
Hb: Haemoglobin
HDS: HIV Dementia Scale
HDS-IT: Italian version of the HDS
HIV: Human Immunodeficiency Virus
IADLs: Instrumental Activities of Daily Living
IHDS: International HIV Dementia Scale
INIs: Integrase Inhibitors
IQR: Interquartile ranges
IVDA: Intravenous Drug Users
LDL: Low-Density Lipoprotein
MoCA: Montreal Cognitive Assessment
MSM: Men who have Sex with Men
NNRTIs: Non-Nucleoside Reverse Transcriptase Inhibitors
NRTIs: Nucleoside Reverse Transcriptase Inhibitors
NtRTIs: Nucleotide Reverse Transcriptase Inhibitors
OR: Odds Ratio
PHQ-2: Patient Health Questionnaire-two items
PIs: Protease inhibitors
PLT: Platelet count
PLWHA: People living with HIV/AIDS
WBC: White Blood Cell count
95%CI: 95% Confidence Intervals
